# Nanomedicine Strategies Utilizing Lipid-Based Nanoparticles for Liver Cancer Therapy: Exploring Signaling Pathways and Therapeutic Modalities

**DOI:** 10.34172/apb.2024.061

**Published:** 2024-07-31

**Authors:** Fereshteh Asgharzadeh, Maryam Moradi Binabaj, Sahar Fanoudi, William C. Cho, Yu-jeong Yang, Maryam Azarian, Mehdi Shafiee Ardestani, Nasim Nasiri, Marzieh Ramezani Farani, Yun Suk Huh

**Affiliations:** ^1^Metabolic Syndrome Research Center, Mashhad University of Medical Sciences, Mashhad, Iran.; ^2^Department of Nutrition, Food Sciences and Clinical Biochemistry, School of Medicine, Social Determinants of Health Research Center, Gonabad University of Medical Science, Gonabad, Iran.; ^3^Department of Basic Medical Sciences, Neyshabur University of Medical Sciences, Neyshabur, Iran.; ^4^Department of Clinical Oncology, Queen Elizabeth Hospital, Kowloon, Hong Kong.; ^5^NanoBio High-Tech Materials Research Center, Department of Biological Sciences and Bioengineering, Inha University, Incheon 22212, Republic of Korea.; ^6^Department of Radiology, Charité - Universitätsmedizin Berlin, Berlin, Germany.; ^7^Department of Radiopharmacy, Faculty of Pharmacy, Tehran University of Medical Sciences, Tehran, Iran.; ^8^Department of Cell and Molecular Sciences, Faculty of Biological Sciences, Kharazmi University, Tehran, Iran.

**Keywords:** Liver cancer, Hepatocellular carcinoma, Signaling pathways, Therapeutic approaches, Lipid-based nanoparticles, Nanomedicine

## Abstract

Liver cancer, specifically hepatocellular carcinoma (HCC), is the second leading cause of cancer-related deaths, following pancreatic cancer. The 5-year overall survival rate for HCC remains relatively low. Currently, there are multiple treatment options available for HCC, including systemic drugs, minimally invasive local therapies such as radiofrequency ablation, transarterial chemoembolization (TACE), and arterial radioembolization (TARE), as well as surgical interventions like liver resection or transplantation. However, the effectiveness of drug delivery to the cancerous liver is hindered by pathophysiological changes in the organ. In order to address this challenge, lipid-based nanoparticles (LNPs) have emerged as promising platforms for delivering a diverse range of therapeutic drugs. LNPs offer various structural configurations that enhance their physical stability and enable them to accommodate different types of cargo with varying mechanical properties and degrees of hydrophobicity. In this article, we provide a comprehensive review of the current applications of LNPs in the development of anti-HCC therapies. By examining the existing research, we aim to shed light on the potential future directions and advancements in this field.

## Introduction

 Liver cancer is projected to cause over one million deaths by 2030, according to the World Health Organization (WHO).^1^ The United States has observed a significant 43% increase in liver cancer mortality between 2000 and 2016.^2^ Hepatocellular carcinoma (HCC), the second leading cause of fatal malignancy after pancreatic cancer, has an overall 5-year survival rate of only 18%.^3^ In 2020, primary liver cancers (PLCs) were estimated to account for 830,180 cancer deaths and 905,677 new cases worldwide, ranking them as the third most common diagnosed diseases. The highest incidence rates were reported in Eastern and South-Eastern Asia, as well as Northern and Western Africa.^4^ HCC is the most prevalent type of PLC globally, accounting for nearly 90% of all liver cancer cases,^5^ while cholangiocarcinoma (CCA) is the second most common form. CCA is a highly lethal malignancy with a 5-year survival rate of less than 10%. It is classified into intrahepatic (iCCA), peripheral (pCCA), and distal (dCCA) subtypes based on anatomic location. iCCA is more commonly associated with hepatitis B virus (HBV)-related cirrhosis, while pCCA is closely related to primary sclerosing cholangitis presenting as chronic inflammation.^6^ Other rare liver cancers include juvenile hepatoblastoma, fibrolamellar HCC, and hepatocellular mixed cholangiocarcinoma (HCC-CCA).^7,8^

 HCC and liver cancer rank as the third deadliest disease and the sixth most common diseases worldwide, according to the WHO.^9,10^ Liver cancer primarily affects men, with the highest incidence rates observed in East Asia, North Africa, and Micronesia. Mortality rates are particularly alarming in East Asia, North Africa, and Southeast Asia. More than 80% of HCC cases are attributable to PLCs.^11^ Inflammatory liver disorders such as alcoholism, nonalcoholic fatty liver disease, and viral hepatitis are common causes of HCC.^12,13^ Epigenetic changes and mutations play a role in the development of chronic liver disorders, including HCC, by activating molecular signaling cascades related to cell proliferation and inhibiting apoptosis.^14^ However, delivering drugs to the cancerous liver is challenging due to pathophysiological changes. While normal liver tissue relies on hepatic artery perfusion for blood supply, portal vein perfusion accounts for 80% of the liver’s oxygen supply.^15^ Systemic administration of drugs results in minimal drug penetration due to insufficient portal vein perfusion. HCC exhibits fibrosis-like behavior, characterized by reduced sinus fenestrae, the need for drugs to cross endothelial barriers, the extracellular matrix, and the tumor stromal barrier to reach the cells. This leads to increased angiogenesis, microvascular density, and permeability in the HCC tumor microenvironment.^16^

**Figure 1:**
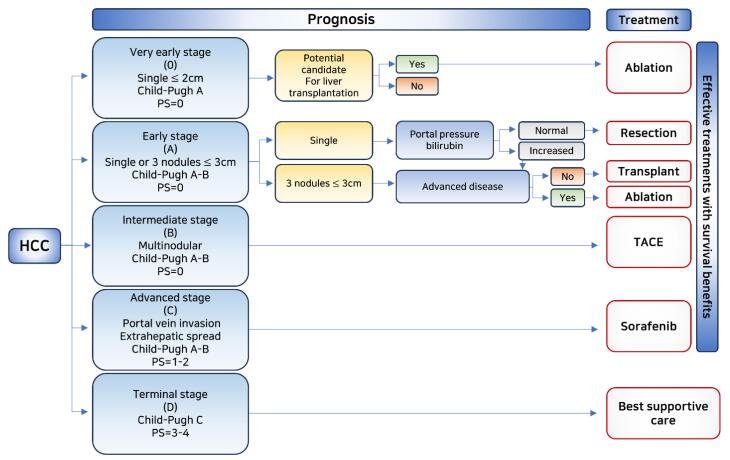
Flowchart depicting prognosis and effective treatment options with survival benefits in HCC

 The enhanced permeability and retention (EPR) effect, coupled with impaired lymphatic drainage, can result in the selective accumulation of macromolecules and nanoparticles in HCC.^17-19^ Several cell proliferation-related receptors and proteins expressed on the surface of HCC cells can be targeted, including ASGPR,^20^ glypican-3,^21^ transferrin receptor,^22^ somatostatin receptor,^23^ glycyrrhetinic acid receptor,^22^ cluster of differentiation 44,^24^ and AF-20 antigen.^25^ Additionally, drugs can be targeted to increase tumor metabolic rate and production of lactic acid and glutathione (GSH).^26^ HCC is characterized by a low pH (pH 6.5) and a strong reducing potential (GSH: 2–10 mM) at the tumor site compared to the extracellular environment and normal tissues (pH 7.4). The pH or redox differential can be utilized to trigger the release of tumor-specific cargoes.^27^ Treatment options for HCC are determined based on staging after diagnosis (Figure 1).

 The initial phases of the illness primarily focus on its management. Hence, primary treatment choices consist of hepatectomy and liver transplantation, if required. In situations where surgical removal is not practicable, radiofrequency (RF) ablation and microwave ablation are suggested. For intermediate stages, the most efficient treatments are chemoembolism, radioembolism, and simple embolism. Despite continuous advancements, systemic therapy for liver disease has been a matter of debate until recently. The development of a targeted multikinase inhibitor, sorafenib, which exhibited a 2.8-month survival advantage over a placebo in 2008, marked its initial development as a treatment option. In 2017, regorafenib, a tyrosine kinase inhibitor, was discovered and replaced despite being an expensive and highly toxic treatment.^28,29^ Lenvatinib is another tyrosine kinase inhibitor recently approved for unresectable HCCs.^30^ Due to the serious side effects of sorafenib and regorafenib, immunotherapy is the chosen first-line therapeutic strategy. Second-line approved therapies include immune checkpoint inhibitors (ICIs) such as nivolumab and pembrolizumab. Previously, phase III studies with immunotherapy treatments failed to show a statistically significant association between progression-free and overall survival rates.

 HCC immunotherapy encounters a significant obstacle as there is currently no delivery technology capable of precisely targeting therapeutic agents to their intended destinations. To address this challenge, lipid-based nanoparticles (LBNPs), for example, liposomes and lipid nanoparticles (LNPs), have been created as potential platforms for delivering various therapeutic drugs.^31,32^ Compared to other delivery methods, LBNPs are superior because they have less systemic toxicity and are clinically more effective than polymeric and inorganic nanoparticles since they are readily soluble in water.^33^

 Lipid-based nanoparticles are the most prevalent type of nanomedicine approved by the US Food and Drug Administration (FDA). These nanoparticles come in a variety of structural configurations that improve their physical stability and their ability to accommodate cargo with varying mechanical properties and degrees of hydrophobicity. The original liposome formulation, created in the 1960s and 1970s for delivering active pharmaceutical ingredients, consisted of a blue inner core surrounded by lipid bilayers. Subsequently, LNPs adopted a micelle-like shape like liposomes but with different lipid structures. It has been demonstrated that the more complex internal lipid architecture of LNPs makes them better suited for encapsulating genetic cargo.^34^ These droplets are composed of nanodroplets that mix the phases of oil and water. Unlike water-in-oil nanoemulsions (NEs), oil-in-water NEs, as an alternative to liposomes and LNPs, have micelle-like structures but are coated with oil droplets.^35^ NEs are less thermodynamically stable than liposomes and LNPs, causing them to undergo phase separation during long-term storage. To improve the kinetic stability of NEs and ensure their efficacy with hydrophobic drugs, a surfactant and lipid adjuvant can be used.^36^ Throughout this article, we reviewed the current applications of LNPs in the development of anti-HCC therapies, which may shed light on how the field may develop in the future.

## Development (signaling pathways)

 The development of liver cancer stem cells (LCSCs) and the preservation of their characteristics during the liver cancer process involve several signaling pathways, including Wnt/Catenin, Hippo, IL-6/STAT3, MAPK, and Notch pathways. The most prominent signaling systems involved in maintaining the stemness of HCC cells are the Wnt/Catenin and IL-6/STAT3 pathways. Somatic mutations in Wnt-related pathway genes are commonly observed in HCC. Additionally, activation of STAT3 frequently occurs in HCC and can transactivate certain genes expressions, such as NANOG, OCT4, and SOX2, which are transcription factors that stimulate the growth of LCSCs.^37^ Interleukin (IL)-6, which is produced by immune cells and hepatocytes, is an upstream regulator of STAT3.^38,39^ The function of STAT3 in different human cancers has been highlighted, and it is an oncogenic factor with a versatile function in accelerating tumorigenesis and the development of drug resistance.^40-43^

 While chronic liver inflammation is frequently linked to the development of HCC, it is worth noting that hepatocytes producing IL-6 may lead to the autocrine activation of STAT3 in hepatocytes. This effect can trigger the reprogramming of liver cells into stem cells, ultimately leading to the development of liver cancer. Furthermore, the tumor suppressor pathway hypo-signaling limits the proliferation of mature stem cells as well as progenitor cells.^44^ When Hippo signaling is activated, the transcriptional coactivators Yes-associated protein 1 (YAP) and transcriptional coactivator with PDZ-binding motif (TAZ) are phosphorylated, leading them to stay in the cytoplasm. This event stops TEA family nuclear transcription factors (TEAD) from interacting with YAP and TAZ, hence suppressing gene expression. Studies have shown that suppression of TAZ expression slows the development of HCC cells. It can also result in the compensatory upregulation of YAP, enhance tumor cell chemoresistance, and increase the expression of CD90, a unique cancer stem cell (CSC) marker for HCC. As YAP and TAZ have different functional roles, simultaneous inhibition of both transcriptional activators is crucial when aiming for the Hippo signaling pathway.^45^ A dysgene and metalloprotease must cleave NOTCH for cancer progression in CSCs (ADAM). NOTCH (NICD) signaling is initiated by inducible nitric oxide synthase (iNOS) via ADAM17/transarterial chemoembolization (TACE) activation, resulting in an active NOTCH intracellular domain.^46^ Interestingly, when three members of the retinoblastoma (RB) family—RB, p107, and p130—were not expressed in the liver, the NOTCH pathway was activated, leading to reduced HCC formation. This finding shows the complex role of NOTCH in the development of liver cancer.^47^

 Liver cirrhosis, which is a leading cause of HCC, involves the crucial role of liver stellate cells or activated fibroblasts. These cells release hepatocyte growth factor (HGF), which can activate the transcription factor FRA1, inducing HEY1 expression in hepatocytes by binding to their c-MET receptor. HEY1 is a downstream target of the NOTCH signaling pathway, and its increased expression may result in an increase in the number of LCSCs and the chemoresistance of hepatocytes, thereby promoting HCC development.^48^ The protein arginine methyltransferase 6 (PRMT6) is methylated and linked to arginine-100 (R100) of c-Raf to inhibit MAPK signaling. When PRMT6 is inhibited, MAPK signaling is stimulated, improving the stemness of CD133^+^ LCSCs.^49^ About 80 to 90% of HCC patients have cirrhosis due to long-term inflammation of the liver.^50^ The primary initiator of inflammation associated with liver cancer is the death of epithelial cells. Tumor necrosis factor (TNF)-α, signal transducer and activator of transcription 3 (STAT3), IL-6, C-Jun N-terminal kinase (JNK), nuclear factor kappa B (NF-κB), innate and adaptive immune signaling, and immunity are examples of pathways that contribute to inflammation-mediated hepatocarcinogenesis.^51^

## Risk factors

 Males have a higher incidence of HCC than females, with a two-fold increased risk. The incidence of liver cancer increases with cirrhosis from any cause, ranging from 2 to 4 percent per year. The risk varies depending on the underlying cause, region, age, gender, and the extent of liver damage.^52^ Worldwide, HCC is mainly caused by HBV infection. Although vaccination against HBV has reduced the incidence of liver cancer,^53^ many unvaccinated individuals are still at risk of developing liver cancer and HBV infection (257 million in 2015), primarily in Asia and sub-Saharan Africa.^54^ Moreover, individuals with HBV infection who have a specific mutation in TP53 at position 249 (RS) are more prone to developing HCC. Conversely, in Western countries and Japan, HCV infection is the primary cause of liver cancer. HBV infection has a direct carcinogenic impact regardless of the severity of underlying liver fibrosis,^55^ whereas liver cancer is uncommon in HCV-infected individuals without extensive fibrosis. Non-alcoholic fatty liver disease (NAFLD) is increasing the prevalence of HCC worldwide. In the United States, this rate is expected to rise by 122% between 2016 and 2030.^56^ Liver cancer is commonly caused by alcoholic cirrhosis. Additionally, HCC can arise due to concurrent smoking and human immunodeficiency virus (HIV) infection. While antiviral medications significantly reduce the likelihood of developing HCC in these individuals, the risk cannot be completely eliminated.^57^ The use of direct-acting antiviral drugs reduces the risk of liver cancer in HCV-infected patients.^58^

 Currently, the primary global risk factors for HCC are HBV and HCV. However, the prevalence of these conditions is declining due to improved care and vaccination efforts. In contrast, the incidence of HCC due to NAFLD/non-alcoholic steatohepatitis (NASH) is rising and could soon surpass viral causes as the leading risk factor. Other significant risk factors include excessive alcohol consumption, exposure to AFB1, obesity, and diabetes. Therefore, greater efforts should be made to combat obesity and diabetes to reduce the incidence of NAFLD. Additionally, more effective strategies to reduce alcohol consumption and control mycotoxin contamination are necessary.^59^

## Staging systems

 HCC should be staged because proper staging leads to treatment recommendations and an accurate prognosis. There are some new HCC staging systems that have emerged in different countries, but none of them are widely recognized or allow for the comparison of different treatment methods. Some clinical staging systems for HCC include the CLIP (Cancer of the Liver Italian Programme) score, MESIAH (Model to Estimate Survival in Ambulatory HCC Patients) score, ITA.LI.CA (Italian Liver Cancer) score, BCLC (Barcelona Clinic Liver Cancer) staging, HKLC (Hong Kong Liver Cancer) staging, and the Alberta algorithm.^60^ Since most patients with HCC also have concurrent liver disease, the benefits of tumor therapy must be evaluated against the risks of medical therapy in individuals with cirrhosis.

**Figure 2:**
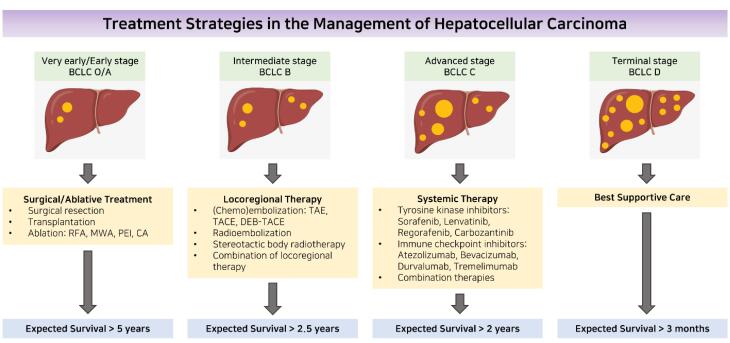
Managing hepatocellular carcinoma through a treatment strategy. Abbreviations: BCLC stands for Barcelona Clinic Liver Cancer; CA stands for cryoablation; DEB-TACE stands for drug-eluting beads transarterial chemoembolization; MWA stands for microwave ablation; PEI stands for percutaneous ethanol injection; RFA stands for radiofrequency ablation; TACE stands for transarterial chemoembolization; and TAE stands for transarterial embolization.^67^ Reprinted with permission from Kung and Ng^67^ (Copyright 2022, OAE Publishing Inc)

 The comprehensive management of liver cancer involves various disciplines such as hepatology, hepatobiliary surgery, pathology, oncology, radiology (both diagnostic and interventional), and specialized nursing. To determine the prognosis of patients, a staging system must consider the extent of liver dysfunction, tumor burden, and performance status. The BCLC staging system, introduced in 1999,^61^ is the most widely used.^62^ This grading approach is recognized as a standard for the development of clinical trials in HCC and is supported by clinical practice guidelines.^63,64^ The algorithm categorizes patients into five stages—very early, early, intermediate, advanced, or late stage—and provides stage-based treatment recommendations (Figure 2). Other staging methods, such as the CLIP,^65^ and the HKLC staging system,^66^ are available but are limited to certain geographic areas. Cross-sectional imaging is used to assess tumor burden, considering factors such as size, number of nodules, and the presence of macrovascular tumor invasion or extrahepatic spread.

## Diagnosis methods

 Imaging techniques can be used in cirrhotic patients to identify HCC. This is because malignant nodules are typically supplied by the hepatic arteries, while benign lesions like regenerative and dysplastic nodules are supplied by branches of the portal system.^68^ Contrast-enhanced CT or magnetic resonance imaging (MRI) scans can reveal the characteristic changes caused by HCC. These changes are seen as hypervascularity during the arterial phase of the scan, followed by hypoperfusion during the portal or delayed phases. This pattern has a sensitivity ranging from 66% to 82% and a specificity of over 90% in detecting HCC in individuals with cirrhosis and nodule diameters larger than 1 cm.^69^ In specialized centers, particularly in Europe,^63^ and Asia,^70^ contrast-enhanced ultrasound is also used to characterize hepatic nodules, although its precise diagnostic performance is still under investigation. For individuals without cirrhosis or with indeterminate patterns on imaging, a biopsy should be relied upon for diagnosis. Histological diagnosis can be challenging in patients with small nodules; however, a panel of immunostaining markers, such as heat shock protein 70, glypican 3, and glutamine synthetase, improves the accuracy of diagnosis.^71^ Patients with cirrhosis and nodules smaller than 1 cm should undergo ultrasound monitoring every 3–4 months, and if the nodule size remains stable after 12 months, routine surveillance may be an option.^63^

## Therapeutic approaches

 Currently, there are several treatment options available for HCC, including systemic drugs, minimally invasive local therapies like radiofrequency ablation, TACE, and arterial radioembolization (TARE), as well as surgical options like liver resection or transplantation. The decision for liver transplantation is based on the Milan criteria.^72^ However, it has been suggested that broader selection criteria than Milan should be considered in some cases.^73,74^ Radiofrequency ablation (RFA), cryoablation, and microwave ablation deliver heat directly to the tumor using needle electrodes, effectively killing tumor cells.^75^ Transarterial injection with a combination of lipiodol, chemotherapeutic medicines, gel foam, or microspheres (traditional TACE), drug-eluting beads (DEB-TACE), or yttrium-99 (TARE) promotes tumor necrosis by reaching the tumor site, as HCC is sustained by blood from the hepatic artery.^76^ Selective internal radiation therapy (SIRT) eliminates tumor cells by generating beta rays.^77^ For patients with advanced-stage HCC, sorafenib, an oral tyrosine kinase inhibitor that targets the fibrosarcoma (Raf)/mitogen-activated protein kinase (MEK)/extracellular signal-regulated kinase (ERK) pathway, vascular endothelial growth factor receptor (VEGFR), and platelet-derived growth factor receptor (PDGFR), is recommended and demonstrated.^78,79^

 Between 2017 and 2019, new first- and second-line systemic therapies targeting tyrosine kinase receptors were developed: regorafenib (a multikinase inhibitor with a mechanism similar to sorafenib),^28^ lenvatinib (a multikinase inhibitor that targets VEGFR 1-3, fibroblast growth factor receptors (FGFR) 1-4, PDGFR α, RET, and KIT),^80^ cabozantinib (a tyrosine kinase inhibitor that targets MET, VEGFR2, and RET),^81^ and ramucirumab (a VEGFR2 antagonist).^82,83^ Nivolumab, another programmed cell death protein-1 (PD-1) inhibitor, was authorized in 2017 as second-line therapy for advanced HCC.^84^ Although new antiviral agents for HBV replication, such as tenofovir and entecavir, inhibit HBV replication, they do not completely eliminate the risk of HCC in people with cirrhosis.^85^ On the other hand, antiviral drugs for HCV, such as sofosbuvir, daclatasvir, ledipasvir, and ribavirin, have shown efficacy in preventing irreversible hepatic decompensation and reducing tumor recurrence after curative therapy.^86^

 The increasing incidence of HCC caused by NASH, particularly in the Indian subcontinent,^87^ requires careful monitoring of HCC progression in patients with NAFLD, as well as the promotion of lipid-lowering medications such as statins and lifestyle adjustments to manage metabolic syndrome. New medications, such as elafibranor, obeticholic acid, cenicriviroc, liraglutide, and aramacular, have the potential to treat NASH and limit the development of HCC. However, their efficacy needs to be studied in future clinical research.^88,89^

 Nanotechnology has the potential to enhance the activity of drugs and destroying cancer cells.^90,91^ Its application can facilitate the development or improvement of treatments that result in better outcomes for neoplastic diseases. This could involve optimizing medication levels and dosages or using tissue-specific delivery systems to target specific locations, reducing the risk of systemic toxicity and unwanted effects.^92^ The use of nanotechnology may revolutionize current approaches to combination therapy by enhancing pharmacokinetic, permeation, and retention profiles, resulting in reduced side effects.^93,94^ Nanoparticle approaches in medicine pave the way for a promising future through therapeutic regimens that combine multiple substances to enhance the effects of drugs.^95,96^ The use of nanotechnology has been explored to enhance drug delivery and efficacy in liver cancer treatment. One potential application is the development of different drug delivery systems using nanocarriers, which can reduce the required drug doses, increase the therapeutic index, minimize the risk of systemic toxicity, enable sustained drug release over days after a single dose, and enhance the selective targeting of liver cancer cells (Table 1).

**Table 1:** Advantage and disadvantage of lipid nanoparticles

**LBNPs**	**Advantages**	**Disadvantage**	**References**
**Figure:** 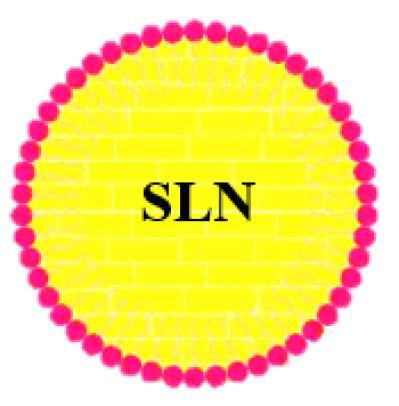	Biologically compatible Organic solvent-free (green synthesis) Scalable and reproducible production method	The perfect crystalline structure reduces encapsulation efficiency. High drug expulsion	^97,98^
**Figure:** 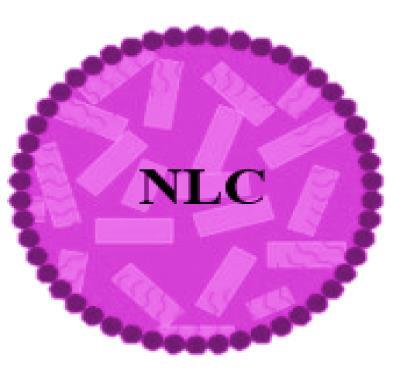	Efficient encapsulation Expulsion of drugs is low	The matrix structure may have cytotoxic effects. Irritating action of some surfactants	^99,100^
**Figure:** 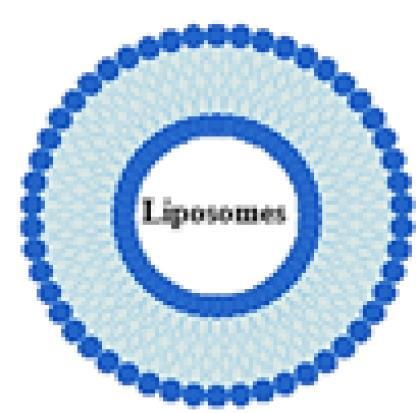	Biologically compatible Biologically degradable Immunogenetically inactive A low level of toxicity	Production costs are high Leakage of drug substances Insufficient half-life Use of phospholipids may result in oxidation and hydrolysis	^100,101^
**Figure:** 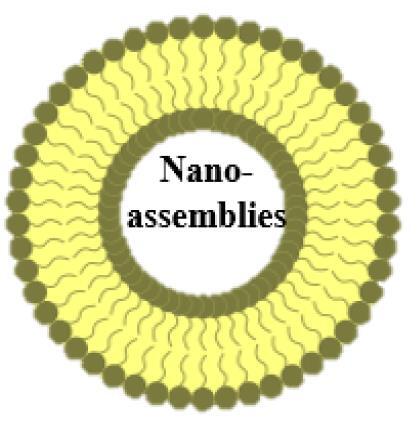	Synthesis in a hurry Dispersibility is simple Economies of scale	Controlling particle size is difficult Scaling up can be challenging Stability of the product over a short period	^102^
**Figure:** 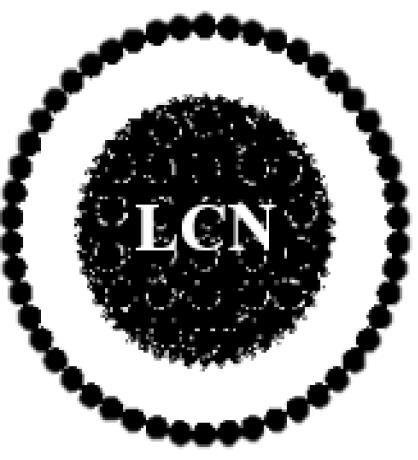	Biological compatibility Maintaining structural integrity Targeting molecules can be conjugated with flexibility	The manufacturing process consisting of multiple steps The scaling up process is challenging	^103,104^
**Figure:** 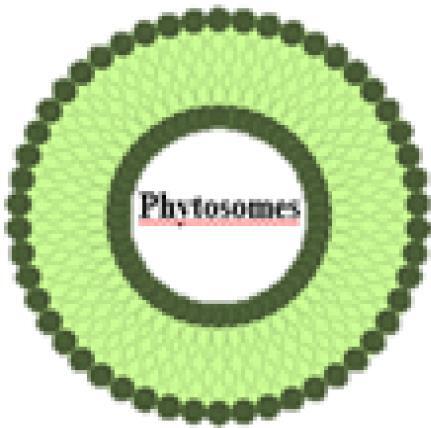	Absorption enhancement An extremely low toxicity level	Leaching caused by phytochemicals Insufficient concentration of the drug	^105,106^
**Figure:** 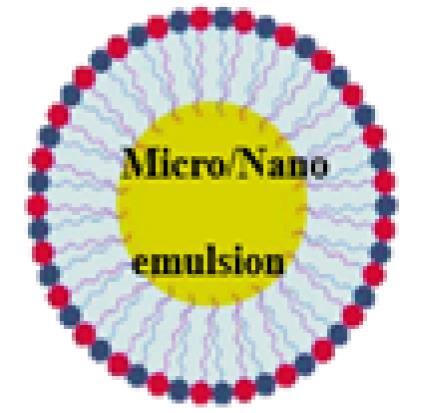	Assemblable Cellular membrane penetration is high An excellent rate of absorption	An excessive concentration of surfactants Possibly separate phases	^107,108^

Abbreviations: SLNs, solid lipid nanoparticles; NLCs, nanostructured lipid carriers; LCNs, lipid-coated nanoparticles

## Evaluating and summarizing different studies on the application of LBNPs in HCC

 LBNP technology is currently being explored as a potential treatment for HCC. Table 2 presents published reports on studies investigating the use of LBNPs for HCC treatment. However, the physicochemical properties of drugs often limit the effectiveness of HCC treatments. Chemotherapy and targeted drugs like sorafenib have minimal impact on patient survival, while radiotherapy is frequently ineffective. To improve treatment efficacy and patient survival, some nanoplatforms have been combined with drugs. For instance, in liver cancer treatment, cubosomes and NLCs loaded with 5-FU and PTX have been utilized. This treatment increases the accumulation of 5-FU in the liver by preventing its rapid degradation by enzymes. Additionally, PTX-loaded NLCs enhance the plasma persistence and aggregation of Intaxelfi, the commercial formulation.^109,110^ Furthemore, a dual treatment system consisting of sorafenib and superparamagnetic iron oxide nanoparticles (SPIONs) loaded into SLNs has been used to treat HepG2 cells.^111^ The antitumor strategies for liver cancer are further enhanced by the development and testing of new nanoformulations. Two new MEs have been developed by Lin et al^112^ and Qu et al^113^: (1) an ME made from soybeans loaded with curcumin (CUR); and (2) an ME made from Coix seed ingredients (C-ME) modified to target the overexpressed asialoglycoprotein receptor.

**Table 2:** Clinical trials for lipid-based nanoparticles (LBNPs) for HCC treatment.

**Stages of the clinical trial**	**Lipid-based NPs**	**Drugs that have been loaded**	**LBNPs target**	**Reference**
Phase 3	Liposomes that respond to temperature	Dox	Release in response to stimulus	NCT00617981
Phase 3	Liposomes that respond to temperature	Dox	Release in response to stimulus	NCT02112656
Phase 1b/2	Nanoparticles containing lipids	Small interfering RNA oligonucleotide	The development of gene therapies targeted at the liver	NCT02314052
Phase 1/2	The combination of glycyrrhizin and gemcitabine (Gem)	Gem	Targeting with an active approach	NCT02449109
Phase 1	Liposome	Mitoxantrone hydrochloride	An approach that is passive	NCT04331743
Phase 3	Liposomes that respond to temperature	Dox	Release in response to stimulus	NCT02112656
Phase 1	Liposomes that respond to temperature	Dox	Release in response to stimulus	NCT00441376
Phase 1	Liposome	RNA with double strands	The development of gene therapies targeted at the liver	NCT02716012
Phase 1/2	Liposome	Aroplatin	An approach that is passive	NCT00057395
Phase 2	Liposomes with pegylation	Dox	An approach that is passive	NCT00003296
Phase 2	Liposome	Irinotecan	An approach that is passive	NCT03044587
Phase 1	Liposomal mimic	miR-34a	The development of gene therapies targeted at the liver	NCT01829971

 At concentrations of 15 mM, toxicity was mainly observed in HepG2 cells, but at higher concentrations, animals with HepG2 tumor xenografts showed increased internalization and toxicity. Additionally, Hu et al^114^ developed a chemotherapeutic and photothermal polyethylene with doxorubicin (Dox) and indocyanine green (ICG). Combining this approach with near-infrared (NIR) laser irradiation completely inhibited H22 tumor models.

## Concluding and future perspectives

 Current cancer treatments are limited to surgery, radiation therapy, and chemotherapy. However, these procedures carry the risk of damaging normal tissues or incomplete eradication of cancer. Nanotechnology provides tools to directly and selectively target chemotherapy to cancer cells and tumors, guide surgical tumor removal, and enhance the effectiveness of radiotherapy and other existing treatments. These advancements can reduce risks for patients and improve their chances of survival. Based on the findings in this review, lipid-based nanostructures hold promise as candidates in liver cancer therapy. Several reasons contribute to the popularity of lipidic nanoparticles, such as their stability, biocompatibility, and decreased undesirable side effects. The cytotoxic activity of anti-neoplastic agents is significantly enhanced by the use of lipidic nanoparticles. A variety of targeting techniques can further enhance lipid-based nanoparticle efficiency. Antineoplastic agents are more likely to reach tumors through targeted approaches, which minimizes their effect on normal tissues. Increasing the effectiveness of anti-neoplastic agents by using lipid-based nanoparticles holds great promise. However, there is still room for improvement in order to increase the number of clinically approved medications. A particular emphasis should be placed on reducing the potential risk of toxicity. It is important to find alternatives to cationic lipids, which may have toxic effects, and to develop solutions that can achieve the positive effects of lipidic nanoparticles without the associated risk of toxicity. This will help to enhance the safety profile of the formulations. Additionally, the methods of preparation used should be a focus for further advancement. The use of novel, environmentally friendly methods that do not rely on organic solvents will not only improve scalability and reduce production costs, but also eliminate the risk of toxic residuals.

## Conclusion

 LBNPs have gained significant traction in both preclinical and clinical settings for drug delivery applications, demonstrating superior advantages compared to other delivery methods. The approval of numerous LBNPs for clinical use underscores their efficacy. The innovation in lipid design and advanced formulations of LBNPs holds the potential for expanding their application in drug delivery. However, a key challenge remains in achieving drug specificity. Despite efforts to develop targeted delivery approaches, practical implementation in clinical settings lags. Various strategies have been investigated to target the effective delivery of LBNPs. The choice of lipid composition influences the biodistribution of medications, with DSPC-based LBNPs showing a predilection for the spleen, while DOPE-based LBNPs tend to accumulate in the liver. Future enhancements in LBNP design for drug delivery will benefit from a comprehensive understanding of structure-function relationships and lipid chemistry. The integration of cutting-edge technologies such as machine learning and meta-data analysis in research publications is anticipated to provide valuable insights for optimizing LBNP design in the future.

## Acknowledgments

 This research was supported by the Brain Pool program funded by the Ministry of Science and ICT through the National Research Foundation of Korea (Grant Number: 1244 2022H1D3A2A02085952). This research was also supported by the National Research Foundation of Korea (NRF) grant funded by the Korea government (MSIT) (No. 2022M3J7A1062940 and RS-2023-00240052).

## Competing Interests

 The authors declare that they have no known competing financial interests or personal relationships that could have appeared to influence the work reported in this paper.

## Ethical Approval

 Not applicable.
